# The redox status of salinity-stressed *Chenopodium quinoa* under salicylic acid and sodium nitroprusside treatments

**DOI:** 10.3389/fpls.2022.1030938

**Published:** 2022-11-01

**Authors:** Shokoofeh Hajihashemi, Omolbanin Jahantigh, Sahira Alboghobeish

**Affiliations:** Plant Biology Department, Faculty of Science, Behbahan Khatam Alanbia University of Technology, Khuzestan, Iran

**Keywords:** antioxidants, membrane integrity, nitric oxide, osmoperotectants, photosynthetic pigments, quinoa

## Abstract

Spreading the cultivation of crops with high nutritional values such as quinoa demands a wide area of research to overcome the adverse effects of environmental stress. This study aimed at investigating the role of salicylic acid (SA) and sodium nitroprusside (SNP) as a nitric oxide donor, priming at improving the antioxidant defense systems in boosting salinity tolerance in *Chenopodium* quinoa. These two treatments, SA (0.1 mM) and SNP (0.2 mM), individually or in combination, significantly improved the function of both enzymatic and non-enzymatic antioxidants. SA and SNP priming significantly reduced superoxide dismutase activity, which was accompanied by a significant decrease in hydrogen peroxide accumulation under salinity stress (100 mM NaCl). The SA and SNP treatment increased the activity of enzymatic antioxidants (e.g., catalase, ascorbate peroxidase, peroxidase, and glutathione reductase) and the accumulation of non-enzymatic antioxidants (e.g. ascorbate–glutathione pools, α-tocopherol, phenols, flavonoids, anthocyanins, and carotenoids) to suppress the oxidative stress induced by salinity stress. Under SA and SNP treatment, the upregulation of antioxidant mechanisms induced a significant increase in chlorophyll florescence, chlorophylls, carotenoids, and proteins, as well as a significant reduction in the malondialdehyde content in salinity-stressed plants. In addition, the foliar application of SA or/and SNP led to a significant increase in the accumulation of osmoprotectant molecules of sugars and proline to overcome osmotic stress induced by salinity stress. In conclusion, SA and SNP priming can effectively combat salinity stress through improving the redox status of plants.

## Introduction

Quinoa (*Chenopodium quinoa* Willd.) is an annual dicotyledonous herbaceous crop in the Amaranthaceae family with agronomic and nutritional value. In about 7,000 years ago, it was domesticated in the Andean countries of South America, while it is reported to be widely cultured in more than 100 countries by 2021. The main edible part of quinoa is its gluten-free grains with high quantities of proteins, amino acids, minerals, and vitamins. Based on its nutritional value and health importance, it is considered to be a “superfood.” The United Nations General Assembly called 2013 as the “International Year of Quinoa” (Pathan and Siddiqui, 2022). Additionally, its salt, drought, and cold tolerance, in combination with the little requirement to fertilizers and water, has introduced quinoa as a preferable crop for cultivation all over the world (Pathan and Siddiqui, 2022).

An excessive level of salt in the soil or irrigation water limits plant growth and productivity (Mostofa et al., 2015). The plant’s exposure to toxic levels of salt induces generation of excessive quantities of reactive oxygen species (ROS), such as superoxide anion (O_2_^−^), hydroxyl radical (OH), and hydrogen peroxide (H_2_O_2_), which can induce oxidation and malfunction in critical macro- and micromolecules in the plant cells. The induced oxidative stress because of the high accumulation of ROS can be mitigated through a complex series of ROS-scavenging or -detoxifying systems such as enzymatic and/or non-enzymatic antioxidants. Such antioxidant systems can improve the plant’s potency to counteract ROS overaccumulation, so as to protect the cells against oxidative stress. The main antioxidants include ascorbate (AsA)–glutathione cycle enzymes and metabolites, peroxidase (POD), superoxide dismutase (SOD), catalase (CAT), phenolic compounds, and proline to overcome oxidative stress. Lipid peroxidation is one of the common injuries induced by the overaccumulation of ROS because of the degradation of lipids and proteins (Yadu et al., 2017).

Salinity stress is one of the most common environmental stress. Understanding the mechanisms involved in the defense response to stressors is very critical in finding new cues to produce stress tolerance plants. In particular, salicylic acid (SA), as a naturally occurring plant phenolic compound, is introduced as one of the natural plant hormones involved in the plant response to different abiotic stress, such as salinity (Rasheed et al., 2022), drought (Ahmad et al., 2021), and heavy metals (Kaya et al., 2020b). The exogenous application of SA could minimize the adverse effects of salinity stress on the physiological parameters of different plant species (Rasheed et al., 2022). SA is thought to protect the plants against environmental stress by balancing ROS accumulation and stabilizing macromolecules such as lipid membranes, proteins, and photosynthetic pigments (Kaya et al., 2020b).

Signaling molecules such as nitric oxide (NO) are found in all plants and implicated in various physiological, biochemical, and developmental processes, as well as responses to abiotic stress (Hajihashemi et al., 2020b; Kolbert et al., 2021). NO serves as a messenger of stress signals. As a result of its antioxidant capability, NO shows antistress effects and membrane and cell wall–stabilizing abilities. The exogenous application of NO has been proposed as an effective approach to enhance stress tolerance of crops, such as *Oryza sativa*, *C. quinoa*, *Glycine max*, and *Medicago truncatula* (Filippou et al., 2013; Mostofa et al., 2015; Hajihashemi et al., 2020b; Jabeen et al., 2021).

Plants apply various strategies to improve tolerance to environmental stress, while some of them are time-consuming. Priming, acclaimed as preexposure of plant to elicitors, induces “stress memory” to enable quicker or more effective activation of diverse defense systems upon plant exposure to stressor (Aranega-Bou et al., 2014; Xiao et al., 2017). The pretreatment of plants with specific compounds or biological agents can maintain efficient action of defense mechanisms because of a higher content of stress-protective compounds under subsequently encountered stress. In this respect, priming practice can be done at various plant developmental stages or life cycle such as seed, seedling, or young plant (Wiszniewska, 2021). Small signaling compounds such as H_2_O_2_ and NO and the phytohormone SA are the most frequently applied to induce priming effects in plants (Wiszniewska, 2021). Priming factors can either trigger the stressor itself or stimulate the defense mechanisms as a stress predictor (Wiszniewska, 2021).

However, the exogenously applied phytoprotectants such as plant hormones and signaling molecules were used to induce a significant protection in plant species subjected to stress throughout the last decades (Yadu et al., 2017); the protection mechanisms of phytoprotectants priming in alleviating the adverse effect of salinity stress needs to be studied more precisely, particularly in important crops. Because quinoa is an important crop, this study was conducted to investigate the effects of plant priming with SA and sodium nitroprusside (SNP), as an NO donor, and their combinations on maximizing plant stress tolerance. In this context, the enzymatic and non-enzymatic antioxidants, as well as photosynthetic pigments, membrane integrity and osmoprotectant molecules were studied in the present research.

## Materials and methods

### Plant cultivation and treatments

**Figure 1:**
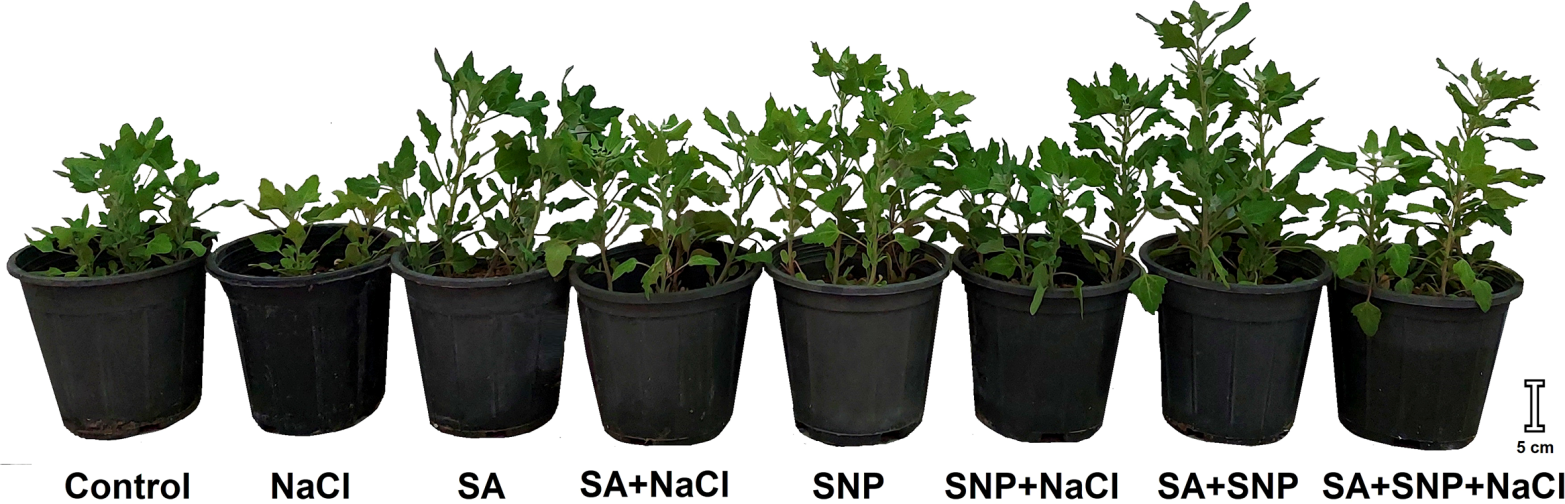
Illustration of *Chenopodium quinoa* under different treatments of salicylic acid (SA) and sodium nitroprusside (SNP), and salinity stress at harvesting stage.

The planting experiment was conducted in Behbahan, Khuzestan, Iran, based on a pot culture using quinoa (*C. quinoa* Willd.) seeds obtained from Pakan Bazr Isfahan, Iran. Ten quinoa seeds were planted per each polyethylene pot containing equal amounts of soil and perlite. The pots were transferred to a room with a 16 h:8 h light/dark cycle and irrigated with tap water every 4 days. At the two leaves stages, four uniform plants were kept per pot to grow further, and extra plants were uprooted. Then, the pots were transferred to open-air conditions. As a completely randomized block in a split-plot design, the foliar treatment and NaCl irrigation were considered as the main plot and subplot, respectively. Each treatment had four replications, that is, four pots per treatment. Each repetition included four plants per pot, so each treatment contained 16 quinoa plants. Before starting the salinity stress, priming was done on young plants with SA and SNP. Some prior characterization of multiple parameters was done to select a meaningful level of SA (0–0.2 mM), SNP (0–0.2 mM), and NaCl (0–200 mM). The results (not shown) revealed that 0.1 mM SA and 0.2 SNP were the most effective on reducing the adverse effects of salinity stress at 100 mM NaCl. In this respect, the 14-day-old plants were foliar sprayed with distilled water (control plants), SA (0.1 mM), SNP (0.2 mM), and a combination of SA and SNP every 5 days. The foliar application was done uniformly, with about 10-ml solution to the plants per pot, as a fine spray using an atomizer. The foliar application lasted for 1 month. One month after the first foliar treatment, the primed pots were divided into two groups of four pots each. The groups were irrigated with distilled water (control) or 100 mM of NaCl solution every 4 days. The plants were grown for further 1 month under salinity stress. At the end of NaCl irrigation regime, the chlorophyll (Chl) florescence was measured, and the plants were harvested for the purpose of doing further analysis. **Figure 1** illustrates the scheme of the treated plants at the harvesting stage. Furthermore, the key physiological and biochemical parameters were studied in the collected plants.

### Analysis of hydrogen peroxide; malondialdehyde; total antioxidant power; and activity of superoxide dismutase, catalase, ascorbate peroxidase, polyphenol oxidase, peroxidase, and glutathione reductase enzymes

The H_2_O_2_ content of fresh leaves was determined based on the Velikova et al. (2000) procedure. The malondialdehyde (MDA) content, as a lipid peroxidation indicator, was measured in the fresh leaves, using thiobarbituric acid, according to the Heath and Packer (1968) method. The fresh leaves were used to quantify the total antioxidant power (FRAP) value, as already described by Szôllôsi and Varga (2002). The activity of enzymes were appraised according to the standard protocols developed for SOD activity (Giannopolitis and Ries, 1977), CAT activity (Aebi, 1984), ascorbate peroxidase (APX) activity (Nakano and Asada, 1987), POD activity (Plewa et al., 1991), and glutathione reductase (GR) activity (Mannervik, 1999).

### Analysis of ascorbate and glutathione metabolites

The fresh leaves were extracted in cold metaphosphoric acid for the purpose of measuring both AsA and glutathione metabolites. The AsA and dehydroascorbate (DHA) contents were quantified according to the protocol of Kampfenkel et al. (1995). The oxidized glutathione (GSSG) and reduced glutathione (GSH) were measured using the procedure of Griffith (1980).

### Analysis of phenols, flavonoids, anthocyanins, and α-tocopherol

A protocol of using Folin’s reagent was used for phenol assay based on the method of Singleton and Rossi (1965). The flavonoid content was measured in the fresh leaves based on the Zhishen et al. (1999) protocol. The anthocyanins of fresh leaves was colorimetrically measured as already described by Wagner (1979). The quantification of α-tocopherol in the fresh leaves was done according to the Baker et al. (1980) method.

### Analysis of chlorophyll fluorescence and photosynthetic pigments

The Chl fluorescence was determined using a portable Chl fluorometer (Pocket PEA, Hansatech Instruments Ltd., King’s Lynn, Norfolk, England). The maximum quantum yield of photosystem II (F_v_/F_m_) and efficiency of both photosystems I and II (PI_abs_) were examined on the surface of fully expanded apical leaves, which were previously adapted in dark for 30 min (Hajihashemi et al., 2018). The photosynthesis pigments of the fresh leaves were appraised by applying the procedure outlined by Wellburn (1994).

### Analysis of water soluble carbohydrates, proteins, proline, and free amino acids

The water soluble carbohydrates (WSC) content was determined according to the phenol–sulfuric acid protocol (Dubois et al., 1956). The total soluble protein content of fresh leaves was determined using the Bradford reagent (Bradford, 1976). The ninhydrin-based colorimetric assay of Bates et al. (1973) was followed to analyze the fresh leaves free proline content. The free amino acid content in the fresh leaves was measured by the procedure as previously optimized by Yemm et al. (1955). The applied methods in this study were described in detail in our earlier literatures (Hajihashemi and Ehsanpour, 2014; Hajihashemi et al., 2018; Hajihashemi et al., 2020b).

### Statistical analyses

The plant culture and treatment were repeated at three consecutive times, every time with four pots containing four plants as one replicate for each treatment. The values presented in figures are the means plus their respective standard error of three independent replicates. The data were subjected to 0.05% level of probability, employing the Tukey’s test using the SPSS statistical (version 24) package. The superscripted letters presented above each column in the figures show the significant differences at *p* ≤ 0.05.

## Results

**Figure 2:**
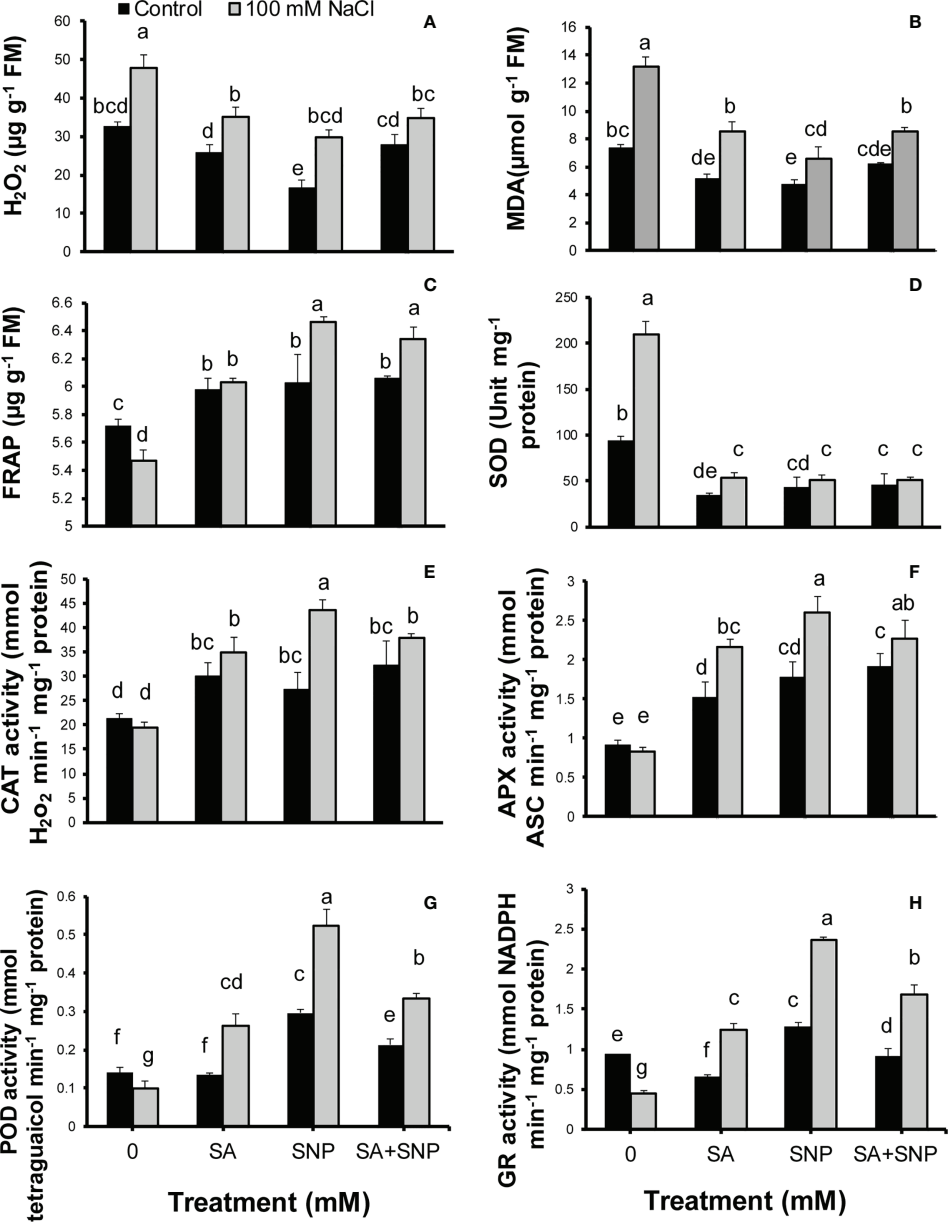
**(A)** H_2_O_2_, **(B)** malondialdehyde (MDA), **(C)** total antioxidant power (FRAP), **(D)** superoxide dismutase activity (SOD), **(E)** catalase activity (CAT), **(F)** ascorbate peroxidase activity (APX), **(G)** peroxidase activity (POD), and **(H)** glutathione reductase activity (GR) in Chenopodium quinoa under different treatments of salicylic acid (SA) and sodium nitroprusside (SNP), and salinity stress. Columns with different letters are significantly different at p < 0.05.

Salinity stress led to oxidative stress as shown by a significant (*p* ≤ 0.05) increase in the H_2_O_2_ and MDA contents, by 32% and 44%, respectively, with respect to the control plants (**Figures 2A**, **B**). The plant priming with both SA and SNP suppressed the accumulation of H_2_O_2_ and MDA in both salt-stressed and non-stressed plants. The SNP treatment induced the largest average reduction in the H_2_O_2_ and MDA contents in the non-stressed plants, by 49% and 35%, respectively, in comparison with the control plants. Under salinity stress, the average values of H_2_O_2_ and MDA showed the highest reduction in response to SNP priming by 38% and 51%, respectively, less than their values in salinity stress without priming. Salinity stress decreased the total antioxidant activity, represented as FRAP, by 5% compared with that in the control plants (**Figure 2C**). The individual foliar application of SA and SNP or their combination improved the FRAP value in both stressed and non-stressed plants. SA, SNP, and SA + SNP priming significantly (*p* ≤ 0.05) increased the FRAP value in the stressed plants by 10%, 15% and 14%, respectively, higher than that in the salt-stressed plants.

The antioxidant system includes enzymatic and non-enzymatic antioxidants. The upregulation of enzymatic and non-enzymatic antioxidants can lead to the reduction of ROS value below the threshold level and the suppression of oxidative stress induced by salinity stress. In this respect, the activity of SOD showed a significant (*p* ≤ 0.05) increase (55%), the activity of CAT and APX showed no significant changes, whereas the activity of POD and GR significantly (*p* ≤ 0.05) decreased (29% and 51%, respectively) in the salt-stressed plants, as compared with that in the control plants (**Figures 2D–H**). SA, SNP, and SA + SNP priming reversed the adverse effects of salinity stress on the activity of SOD, CAT, APX, POD, and GR. Under salinity stress, the activity of CAT, APX, POD, and GR in the SA-, SNP-, and SA + SNP–treated plants significantly (*p* ≤ 0.05) increased higher than their values in the stressed plants without treatment. Under salinity stress, the greatest increase in the activity of CAT (51%), APX (65%), POD (73%), and GR (60%) was achieved in the SNP-treated plants, with respect to the control plants. The foliar application of SA, SNP, and SA + SNP significantly (*p* ≤ 0.05) decreased the SOD activity in both stressed and non-stressed plants (**Figure 2D**). Under salinity stress, the SOD activity in the SA-, SNP-, and SA + SNP–treated plants decreased by 75%, less than its value in salinity stress alone.

**Figure 3:**
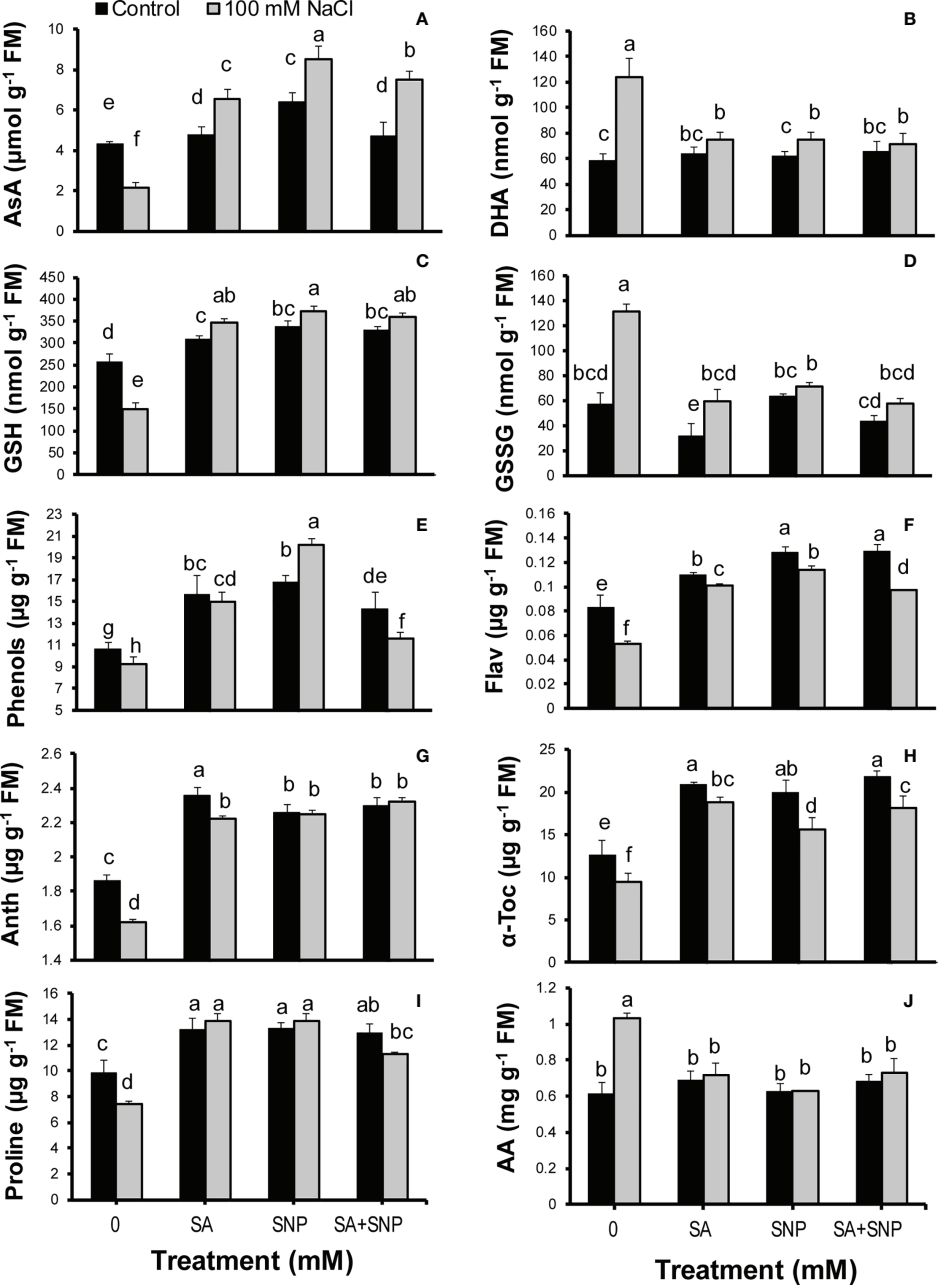
**(A)** Ascorbate (AsA), **(B)** dehydroascorbate (DHA), **(C)** reduced glutathione (GSH), **(D)** oxidized glutathione (GSSG), **(E)** phenols, **(F)** flavonoids (Flav), **(G)** anthocyanins (Anth), **(H)** α-tocopherol (α-Toc), **(I)** proline, and **(J)** total free amino acids (AA) in *Chenopodium quinoa* under different treatments of salicylic acid (SA) and sodium nitroprusside (SNP), and salinity stress. Columns with different letters are significantly different at *p* < 0.05.

To assess the SA and SNP function in tolerance to salinity stress, the redox status of AsA and GSH pools were measured in quinoa plants. Salinity stress significantly (*p* ≤ 0.05) decreased the AsA and GSH contents (50% and 41%, respectively) while significantly (*p* ≤ 0.05) increased the DHA and GSSG contents (53% and 57%, respectively), compared with the control plants (**Figures 3A–D**). SA, SNP, and SA + SNP priming reversed the adverse effects of salinity stress on the AsA and glutathione pools, which was followed by a significant (*p* ≤ 0.05) increase in the AsA and GSH values and a significant (*p* ≤ 0.05) reduction in the DHA and GSSG contents (**Figures 3A–D**). Under salinity stress, the greatest increase in the AsA and GSH contents was achieved in SNP priming, by 75% and 60%, respectively, with respect to their values in salinity stress alone. The values of DHA and GSSG in the stressed plants showed the highest reduction in the SA + SNP treatment by 43% and 56%, respectively, relative to salinity stress alone.

The evaluation of non-photosynthetic pigments revealed a significant (*p* ≤ 0.05) reduction in the phenol, flavonoid and anthocyanin contents in response to NaCl irrigation, by 13%, 36%, and 13%, respectively, as compared with the control plants (**Figures 3E–G**). SA, SNP, and SA + SNP priming significantly (*p* ≤ 0.05) increased the phenol, flavonoid, and anthocyanin contents in both stressed and non-stressed plants. Under stress conditions, the greatest accumulation of phenols and flavonoids was achieved in the SNP treatment by 54% and 53%, respectively, greater than their contents in salinity stress alone. The greatest increase in the anthocyanins in the stressed plants was achieved in the SA + SNP treatment by 30% higher than that in salinity stress alone. The α-tocopherol content, in a similar trend to non-photosynthetic pigments, significantly (*p* ≤ 0.05) decreased in the stressed plants by 24% less than its value in the control plants (**Figure 3H**). The foliar application of SA, SNP, and SA + SNP reversed the adverse effect of salinity stress on α-tocopherol, with the greatest increase observed in the SA treatment, by 49% higher than that in the control plants. The proline content significantly (*p* ≤ 0.05) decreased (24%) in the salt-stressed plants, whereas the amount of total amino acids significantly (*p* ≤ 0.05) increased (40%) in response to salinity stress (**Figures 3I, J**). The foliar application of SA, SNP, and SA + SNP significantly (*p* ≤ 0.05) increased proline accumulation in both stressed and non-stressed plants. Under stress conditions, individual SA and SNP primings were more effective than their combination in increasing the proline content, by 46% higher than that in salinity stress alone. In opposite to proline, the application of SA, SNP, and SA + SNP prevented the changes in the accumulation of amino acids in both stressed and non-stressed plants.

**Figure 4:**
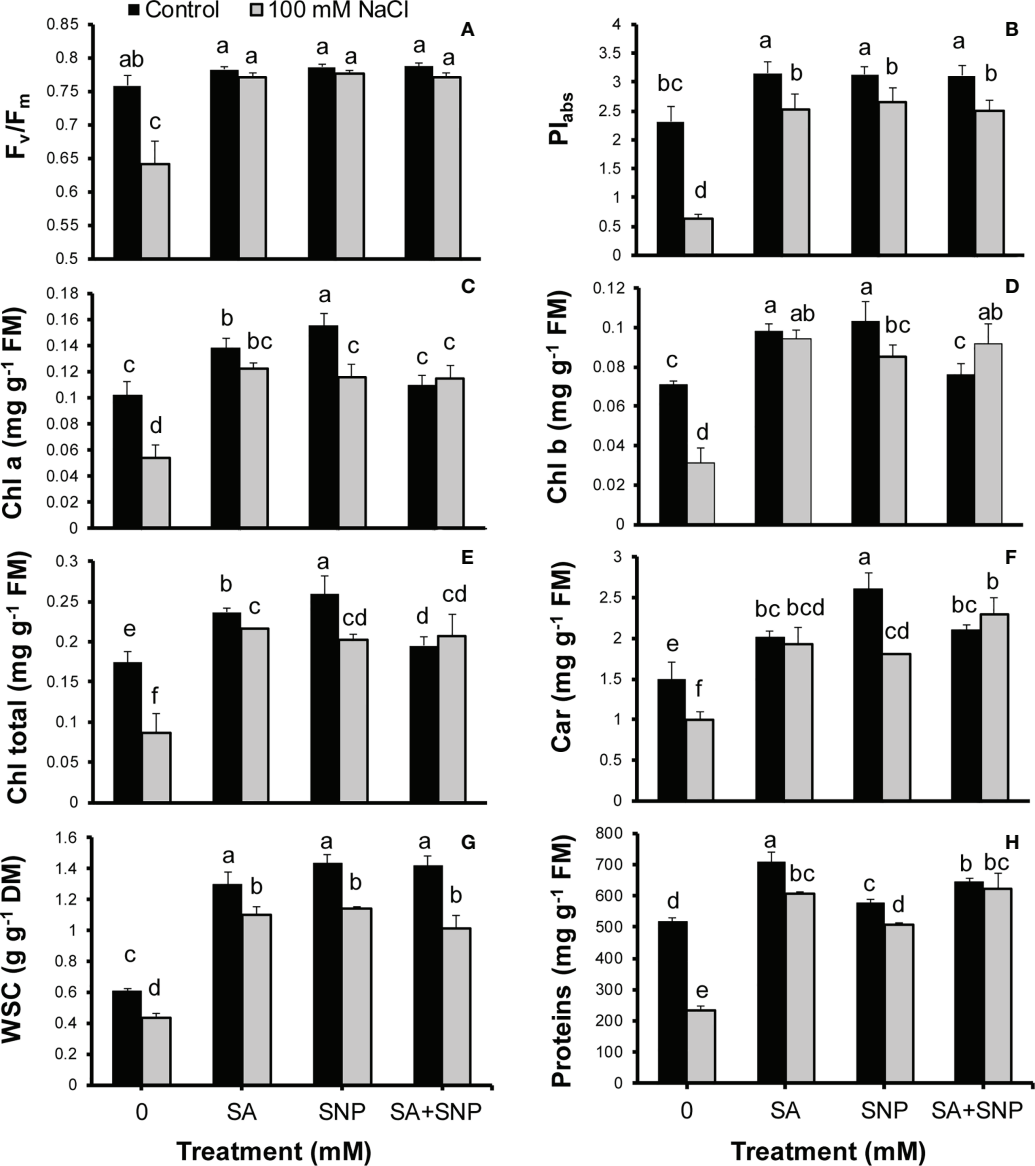
**(A)** Maximum quantum yield of photosystem II (F_v_/F_m_), **(B)** efficiency of both photosystems I and II (PI_abs_), **(C)** chlorophyll a (Chl a), **(D)** Chl b, **(E)** total Chls, **(F)** carotenoids, **(G)** water soluble carbohydrate (WSC), and **(H)** proteins in *Chenopodium quinoa* under different treatments of salicylic acid (SA) and sodium nitroprusside (SNP), and salinity stress. Columns with different letters are significantly different at *p* < 0.05.

Salinity stress suppressed photosynthesis-related traits such as F_v_/F_m_, PI_abs_, Chls a and b, and carotenoids in quinoa plants (**Figures 4A–F**). The F_v_/F_m_ and PI_abs_ values in the salt-stressed plants were by 15% and 72%, respectively, less than their values in the control plants (**Figures 4A, B**). The SA, SNP, and SA + SNP treatments led to a significant (*p* ≤ 0.05) increase in the F_v_/F_m_ and PI_abs_ values as compared with salinity stress alone. In parallel to Chl fluorescence parameters, the Chls a and b, total Chl, and carotenoid values significantly (*p* ≤ 0.05) decreased in the salt-stressed plants by 47%, 56%, 51%, and 34%, respectively, relative to their values in the control plants (**Figures 4C–F**). The foliar application of SA, SNP, and SA + SNP, in the absence or presence of NaCl, promoted a significant (*p* ≤ 0.05) increase in the Chl and carotenoid values. The highest increase in Chls a and b, total Chl, and carotenoid contents was achieved in the SNP treatment by 34%, 31%, 33% and 42%, respectively, higher than them in the control plants.

Along with the observed reduction in the photosynthetic pigments in the salinity-stressed plants, the WSC content also showed a significant (*p* ≤ 0.05) reduction by 28% less than that in the control plants (**Figure 4G**). The SA, SNP, and SA + SNP treatments led to a significant (*p* ≤ 0.05) increse in the WSC content in both streseed and non-stressed plants. The WSC content showed the greatest increse at the SNP application without or with NaCl irrigation by 58% and 47%, respectively, higher than that in the control plants (**Figure 4G**). Furthermore, the protein content significantly (*p* ≤ 0.05) reduced in response to salinity stress by 55% less than that in the control plants (**Figure 4H**). In parallel with the observed increase in the activity of antioxidant enzymes, the foliar application of SA, SNP, and SA + SNP significantly (*p* ≤ 0.05) increased the protein content in both stressed and non-stressed plants (**Figure 4H**). The greatest protein content was observed in the SA treatment by 27% higher than that in the control plants. SA, SNP, and SA + SNP priming in the stressed plants increased the protein content by 61%, 53% and 62%, respectively, relative to that in the stressed plants alone.

## Discussion

Soil salinization with sodium chloride ions induces malfunction in the physiological and biochemical mechanisms due to inducing either osmotic or oxidative stresses, leading to a reduction in the crops yield (Gupta et al., 2018; Gupta et al., 2021). Upon exposure to saline environment, plants employ antioxidants and osmoregulation mechanisms to tolerate salinity stress (Gupta et al., 2021). Two signaling molecules of NO and SA are introduced as robust tools to mitigate the adverse effects of salinity stress through engaging in an array of tasks against salinity-induced oxidative stress (Mostofa et al., 2015; Kaya et al., 2020a; Prakash et al., 2021). SA involves in stress tolerance due to scavenging free radical molecules such as NO and its related molecules, while NO triggers an increase in SA level with the potential of reducing NO-mediated oxidative stress (Prakash et al., 2021). However, the individual roles of NO and SA on enhancing antioxidant systems have been extensively studied (Mostofa et al., 2015; Kaya et al., 2020a; Hajihashemi, 2021; Prakash et al., 2021; Hajihashemi and Jahantigh, 2022); understanding their interactions and associated mechanisms in improving antioxidative systems can highlight future perspectives. This study was designed to evaluate how the antioxidant systems of salt-stressed quinoa plants respond to SA and SNP priming, and the consequence of physiological and biochemical processes was also evaluated to better understand the associated mechanisms toward stress tolerance.

The H_2_O_2_ burst in response to environmental stress plays a critical role in triggering the defense mechanisms in plants to cope oxidative stress. Priming agents such as NO an SA used to stimulate plant antioxidant capacity and to counteract oxidative damages at the early stage of encountering stress (Aranega-Bou et al., 2014; Wiszniewska, 2021).

The results of this study showed that salinity stress induced a significant decrease in the FRAP value accompanied by the high accumulation of H_2_O_2_, which confirms the previous reports based on the negative effects of salinity stress on the antioxidant systems (Mostofa et al., 2015; Hasanuzzaman et al., 2021). In opposite, SA, SNP, and SA + SNP priming promoted a significant increase in the FRAP value in the salinity-stressed quinoa plants, corresponding a significant decrease in H_2_O_2_ accumulation. Under stress conditions, the function of SA + SNP treatment in improving the FRAP value was similar to individual SNP priming and significantly higher than the SA treatment, which declares no antagonistic effect of SA on NO at their applied concentrations in this study. As expected, an increase in the MDA level and a decline in the protein content concomitantly happened with the increase of ROS and reduction of FRAP value in the stressed quinoa plants, which corroborates previous reports (Mostofa et al., 2015; Prakash et al., 2021). The priming of plants with SA, SNP, and SA + SNP prevented the reduction in proteins and the increase in MDA because of salinity stress in a coordinated manner to previous reports (Mostofa et al., 2015; Kaya et al., 2020a; Prakash et al., 2021). Accordingly, the promotion of antioxidant power by SA, SNP, and SA + SNP, represented as an improvement in the FRAP value, was responsible for the observed reduction in ROS accumulation, and the common damages to the lipid and protein molecules in the salinity-stressed quinoa plants.

**Figure 5:**
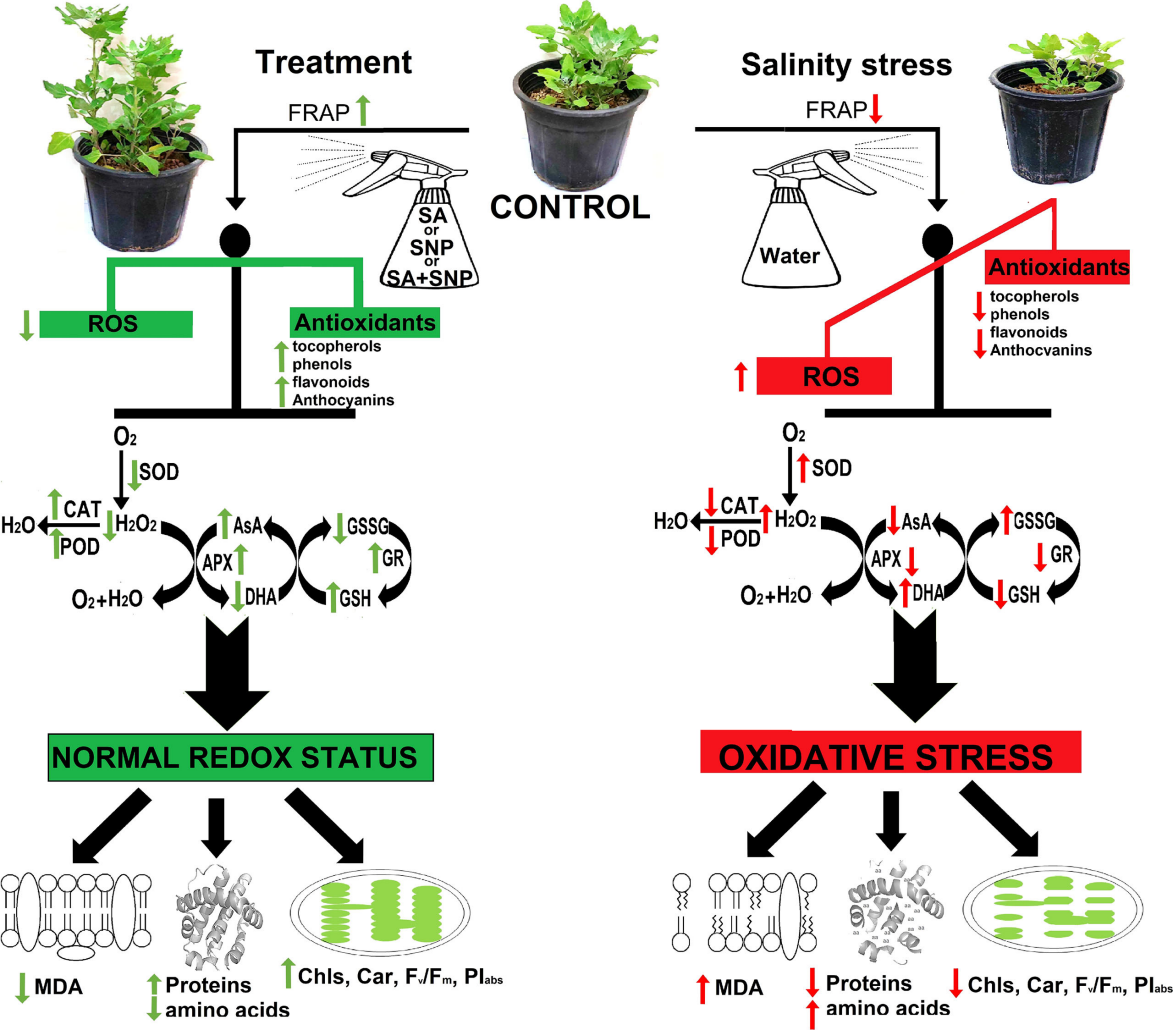
Summary of the most important events that occurred in *Chenopodium quinoa* under different treatments of salicylic acid (SA) and sodium nitroprusside (SNP), and salinity stress.

The promotion of enzymatic and non-enzymatic antioxidant mechanisms was achieved in response to SA, SNP, and SA + SNP priming in the stressed and non-stressed quinoa plants. As illustrated in **Figure 5**, SOD, CAT, APX, POD, and GR are the key ROS scavenging enzymes in plants (Hajihashemi and Ehsanpour, 2014; Hajihashemi, 2021; Kaya et al., 2020b). SA, SNP, and SA + SNP priming reduced H_2_O_2_ accumulation in parallel with a significant decrease in the SOD activity under salinity stress, which approved the previous reports based on the key role of SA and SNP in reducing ROS accumulation (Mostofa et al., 2015; Yadu et al., 2017; Kaya et al., 2020b). In reverse to SOD, the foliar application of SA, SNP, and SA + SNP in the salinity-stressed plants led to a significant increase in the activity of CAT, APX, GR, and POD enzymes, which play a vital role in the H_2_O_2_ detoxification (**Figure 5**) (Mostofa et al., 2015; Yadu et al., 2017). The findings of this study showed that the highest activity of antioxidant enzymes was achieved in the individual SNP treatment. Zottini et al. (2007) demonstrated the role of SA in inducing NO production in *Arabidopsis thaliana* by nitric oxide synthase-like enzymes. Kaya et al. (2020b) has shown that the application of cPTIO, an NO scavenger, upturned the upregulated activity of antioxidant enzymes induced by SA treatment. They have suggested that the accumulation of endogenous NO due to SA treatment involved in inducing antioxidant defense systems (Kaya et al., 2020b). The results of this study approved the previous reports based on the role of SA in modifying the activity of NO-regulated antioxidant enzymes such as SOD, CAT, APX, and GR (Mostofa et al., 2015). Based on the wide involvement of NO in the reduction of oxidative stress (Mostofa et al., 2015), it can be suggested that the salinity tolerance of quinoa in response to both SA and SNP treatment was due to the NO role in suppressing ROS accumulation.

AsA–glutathione cycle is one of the major antioxidant defense pathways in a plant cell with the main aim of detoxifying H_2_O_2_ (Hasanuzzaman et al., 2019). In this cycle, APX decomposes H_2_O_2_ to water with consuming AsA as an electron donor. Then, GR involves in the regeneration of AsA and GSH using NADPH, as the electron donor (Hasanuzzaman et al., 2019). In this study, salinity stress induced a substantial reduction in the AsA and GSH contents, interfering that salinity stress perturbed the whole antioxidant systems. As AsA has prominent responsibility in the redox function metabolism, recycling of AsA from DHA (using GSH) is a necessity to keep the redox balance and a higher total AsA pool (Hasanuzzaman et al., 2019). AsA functions as both a ROS scavenger and an electron donor in various reactions, which leads to be oxidized to DHA. DHA is very unstable and reduces back to AsA; otherwise, it will be lost within minutes. The recycling of AsA can provide its appropriate pool for suppressing oxidative stress in the stressed plant cells (Szarka et al., 2012). In this study, SA and/or SNP priming in the salinity-stressed plants led to the overactivity of APX and GR, along with the changes in equilibrium between AsA and GSH. The achieved increase in the AsA/DHA and GSH/GSSG ratio in response to SA and/or SNP priming was followed with a significant reduction in the H_2_O_2_ content in the salinity-stressed quinoa plants. In line with our findings, Kaya et al. (2020a) acclaimed the potential of combined application of SA plus NO to trigger the AsA–GSH cycle to improve salinity tolerance through suppressing oxidative stress. Despite the association of GR and GSH with AsA regeneration, the overexpression of GR has not led to an improvement in stress tolerance (Szarka et al., 2012). Accordingly, it can be suggested that the observed increase in GR and GSH in response to SA and/or SNP in the stressed plants can provide the reduction state of the AsA pool with coupling of the reactions of the AsA–GSH pathway. Quinoa priming with either SA or SNP is considered advantageous because of its effective response to salinity stress by neutralizing oxidative impairments as a result of enhanced antioxidant systems.

The non-enzymatic antioxidants system includes AsA, GSH, α-tocopherol, flavonoids, phenols, carotenoids, and several osmoprotectants with roles in quenching the toxic by-products of ROS (Gupta and Huang, 2014; Hajihashemi et al., 2020a; Hasanuzzaman et al., 2021). Salinity stress in quinoa plants led to a significant reduction in the non-enzymatic antioxidant molecules such as α-tocopherol, flavonoids, phenols, and carotenoids. AsA and GSH are introduced as the two major hydrophilic antioxidants in the plant cells (Szarka et al., 2012). Tocopherols are lipophilic free radical scavengers with the ability to donate the phenolic hydrogen to lipid free radicals. α-Tocopherol is one of the most abundant antioxidants in leaves. AsA, as one of the most powerful H_2_O_2_ scavengers, maintains the reduced state of α-tocopherol (Szarka et al., 2012). The synergistic antioxidant effect of tocopherol, AsA, and GSH can be an interesting area of the present research. In parallel with AsA and GSH, the foliar application of SA and/or SNP increased α-tocopherol accumulation in the stressed and non-stressed plants. It has already documented that a severe AsA deficiency in chloroplasts led to α-tocopherol loss under stress conditions, which enhanced the importance of the AsA and GSH cycle in recycling reduced α-tocopherol from the tocopheroxyl radical (Szarka et al., 2012; Hasanuzzaman et al., 2019). The reduction of oxidative stress induced by salinity stress in the SA- and/or SNP-treated quinoa plants, represented by the reduction of H_2_O_2_ and membrane lipid peroxidation, increased the belief based on the several fold improvement of the antioxidants of tocopherol, AsA, and GSH in a coordinate manner. In addition, the observed increase in the photosynthetic and non-photosynthetic pigments of carotenoids, phenols, anthocyanins, and flavonoids with antioxidant property in the SA and/or SNP treatment in the salinity-stressed plants provided a strong evidence that the objective of avoiding oxidative damage can be achieved more efficiently by a joint effort of antioxidants.

Excess ROS generation and oxidative stress can lead to hampering photosynthesis, changing enzyme activities, disruption of membranes, and cell death (Hasanuzzaman et al., 2019). The chloroplast is one of the major sources of ROS in plant cells. It is well known that chloroplast, photosystems, Calvin cycle activity, and photosynthesis pathway are the maximum contributor of ROS and oxidative stress under abiotic stress (Hasanuzzaman et al., 2019). In the salinity-stressed plants, enhanced ROS accumulation can lead to damages in the photosynthetic system (Mostofa et al., 2015), which was obvious in this study by a significant reduction in the Chls a and b, F_v_/F_m_, and PI_abs_. The limitation of CO_2_ fixation accompanies a reduction in the ATP and NADPH consumption, which results in a decline in the NADP^+^ content. The depletion of NADP^+^ as the main electron acceptor in photosystem I accelerates the transport of electrons to molecular oxygen resulting in H_2_O_2_ accumulation. The elevated ROS content inhibits the damaged photosystem II repair and results in photoinhibition (Szarka et al., 2012). This may explain the observed reduction in the F_v_/F_m_ and PI_abs_ values in the salinity-stressed quinoa. Several research findings reported about the role of AsA and GSH in improving the Chls and carotenoids levels (Hasanuzzaman et al., 2019), which can explain the observed increase in the Chls, F_v_/F_m_, and PI_abs_ in response to SA and/or SNP application under salinity stress. Wang et al. (2010) have already reported that transgenic plants with a large AsA pool showed lower membrane damage and accumulated a higher level of Chl in the stressed plants. α-Tocopherol accumulation in response to SA and/or SNP priming in the salinity-stressed quinoa plants can be another reason for enhanced ROS scavenging and to avoid oxidative damage to the plastids envelope (Szarka et al., 2012). Overall, the elegant evidence of the interplay between hydrophilic and lipophilic antioxidants has been documented in this study to better understand the early and effective role of the SA and/or NO priming in predicting oxidative stress and hampering oxidative damage to a photosynthetic system.

The overproduction of ROS in cells because of environmental stress is toxic and reactive, which can result in the oxidation of cellular components and damage Chls, proteins, lipids, and DNA (Wang et al., 2010; Hasanuzzaman et al., 2019). The enhanced MDA level in the salinity-stressed quinoa marked the high accumulation of H_2_O_2_ and higher lipid peroxidation than in the control plants. A characteristic feature of MDA accumulation is damages to plant cellular compartment membrane including chloroplasts (Szarka et al., 2012). One response to salinity stress is generation of signaling molecules of SA and NO to combat ROS formation (Gupta et al., 2021). NO can directly react with lipid radicals to prevent lipid oxidation or activate enzymatic and non-enzymatic antioxidants including CAT, APX, GR, POD, and ASA–GSH pathway (Gupta et al., 2021). Besides, there is evidence that tocopherol mediated plant cell protection against lipid peroxidation. Tocopherols are lipophilic molecules with a hydrophobic prenyl tail associated with membrane lipids and a polar chromanol head exposed to membrane surface. Tocopherol synthesis is regulated in plant responses to environmental stress and stress-sensitive hormones such as SA (Noreen and Ashraf, 2010). The parallel enhanced α-tocopherol and stabilized lipid membrane, represented as the reduction of MDA, demonstrated the potential of SA and NO in improving the antioxidant defense system to improve membrane integrity. In line with our results, Szarka et al. (2012) have proposed an interplay between hydrophilic (AsA) and lipophilic (tocopherol) antioxidants to prevent lipid peroxidation under stress conditions.

Under salinity stress conditions, the quinoa plant developed a significant increase in the total amino acids compared with the stress-free condition, and this imbalanced condition triggered reduced protein content. In salinity-tolerant plants, proteins and membrane lipids are protected from oxidative damage through enhanced antioxidant mechanism, which minimizes lipid and protein oxidation while preserving membrane integrity (Gupta et al., 2021). The utilization of SA and/or SNP managed to alleviate the damage to cellular compounds such as proteins through activating the antioxidant systems to scavenge ROS, thereby restricting protein degradation and high amino acid accumulation. Regardless to total amino acids, salinity stress led to a significant reduction in the proline content in quinoa. One of the strategies used to mitigate against salinity stress is the accumulation of proline within the plant cells (Gupta et al., 2021). Under stress conditions, the foliar application of SA and/or SNP resulted in the high accumulation of proline in quinoa. The plant exposure to saline conditions induces a decline in the capacity of water absorbing from soil, resulting in osmotic stress. In order to overcome osmotic stress, the plant cells accumulate osmolytes, such as proline, sugars, organic acids, and polyamines, in favor of osmotic adjustment (Hajihashemi, 2020; Hasanuzzaman et al., 2021; Gupta et al., 2021). In the salinity stress, SA and/or SNP priming increased the proline and sugar contents, suggesting that SA and NO could improve the water potential of cells by enhancing osmolytes, as has earlier been reported in maize and rice (Mostofa et al., 2015; Kaya et al., 2020b). The salinity stress–induced decline in the photosynthetic pigments and photosystems function triggered a significant reduction in the carbohydrate biosynthesis in quinoa, which was reversed by SA and/or SNP priming. As an osmoprotector, it is supposed that sugars can stabilize lipid membranes and proteins through substituting the water molecules in the hydrogen bond formation with phosphate groups of phospholipids and polar residues of polypeptides (Yadu et al., 2017). Overall, plant priming with the relevant molecules of SA and NO motivated various defense mechanisms to improve salinity tolerance in quinoa.

## Conclusion

The complex phenomenon of plant response to SA and NO priming involved dynamic changes in (A) the antioxidant systems including (a) high accumulation of non-enzymatic antioxidants (e.g., AsA, GSH, anthocyanins, flavonoids, phenols, α-tocopherol, and carotenoids) and (b) regulation of enzymatic antioxidants activity (e.g., CAT, APX, POD, GR, and SOD) to combat formation of ROS; (B) osmolytes accumulation (e.g., proline and sugars) to overcome osmotic stress induced by salinity stress; and (C) protecting of cellular compounds against stress-induced damages (e.g., Chls, lipid membrane, and proteins) to modulate the adverse effect of salinity stress in quinoa plants. In conclusion, the results of this study revealed the high potential of relevant molecules of SA and NO in reducing the adverse effect of salinity stress in plants through improving antioxidant defense systems and osmotic adjustment. However, the results reveled that none of SA and SNP at the applied concentrations in this study was superior to the other one. It should also be pointed that the combination of SA and SNP did not multiply their effect. Overall, the scheme of physiological mechanisms induced by the SA and NO priming of quinoa provided a promising strategy in crop production management under stress conditions.

## Data availability statement

The raw data supporting the conclusions of this article will be made available by the authors, without undue reservation.

## Author contributions

SH designed the experiment and wrote the manuscript. SH, OJ and SA conducted the experiment. SH and OJ analyzed the data. All authors contributed to the article and approved the submitted version.

## Conflict of interest

The authors declare that the research was conducted in the absence of any commercial or financial relationships that could be construed as a potential conflict of interest.

## Publisher’s note

All claims expressed in this article are solely those of the authors and do not necessarily represent those of their affiliated organizations, or those of the publisher, the editors and the reviewers. Any product that may be evaluated in this article, or claim that may be made by its manufacturer, is not guaranteed or endorsed by the publisher.
